# Clinician and Patient Characteristics Associated With Lung Cancer Screening Following a Shared Decision-making Visit

**DOI:** 10.1001/jamanetworkopen.2020.21197

**Published:** 2020-10-19

**Authors:** James S. Goodwin, Shuang Li

**Affiliations:** 1 Department of Internal Medicine, University of Texas Medical Branch at Galveston, Texas; 2 Sealy Center on Aging, University of Texas Medical Branch at Galveston

## Abstract

This cohort study explores whether clinician specialty and patient familiarity with the attending clinician are associated with rates of low-dose computerized tomographic lung cancer screening after shared decision-making visits.

## Introduction

The United States Preventive Services Task Force has recommended^1^ and the Centers for Medicare & Medicaid Services has mandated^2^ a separate visit in which a clinician and the patient participate in shared decision-making (SDM) prior to deciding on low-dose computerized tomographic (LDCT) screening for lung cancer.

The purpose of this study was to determine whether the rate of subsequent LDCT screening is associated with the type of clinician visited and with whether the clinician had a prior relationship with the patient.

## Methods

This cohort study used a 20% random sample of national Medicare data to determine enrollees aged 55 to 80 years who had a separate visit for SDM (*Current Procedural Terminology* [*CPT*] code G0296) from January 1, 2016, to September 30, 2018, with complete insurance enrollment 1 year prior. We assessed the clinician specialty from the carrier file and whether that clinician had submitted a bill for services to that patient in the prior 12 months. We assessed receipt of LDCT screening for lung cancer (*CPT* codes 0297 and S8032) in the following 3 months. We have reported observed rates and odds ratios of receipt of LDCT from generalized linear mixed models with and without clinician characteristics.

The University of Texas Medical Branch institutional review board approved the study, and exempted it from the requirement for informed consent because participants were not identifiable in the data. We followed the Strengthening the Reporting of Observational Studies in Epidemiology (STROBE) reporting guideline. All analyses were performed with SAS Enterprise version 7.1 (SAS Institute) at the CMS Virtual Research Data Center.

## Results

From January 1, 2016, to September 30, 2018, 11 699 enrollees in the cohort had a separate visit for SDM. Of the patients who underwent a subsequent LDCT, 91.6% (4652 of 5081 patients) had done so within 3 months (Figure), so we used that cutoff for the remaining analyses. An SDM visit was followed within 3 months by LDCT in 7522 enrollees (64.3%). The Table reports the unadjusted rates and adjusted odds ratios for receipt of LDCT by enrollee characteristics and type of clinician conducting the SDM.

**Figure.**
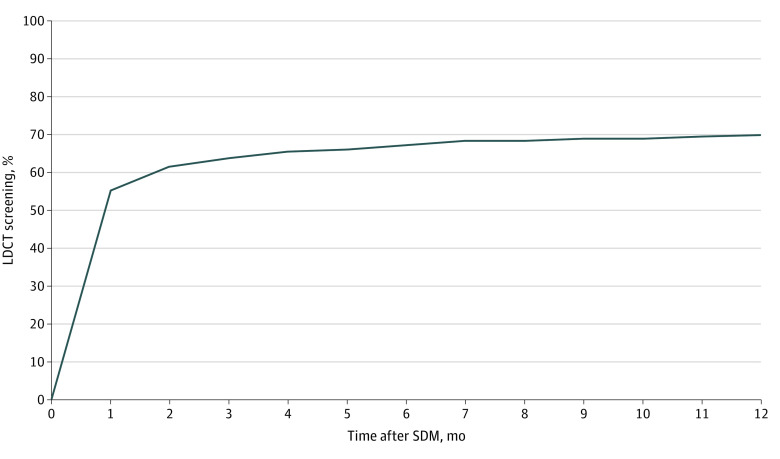
Shared Decision-making (SDM) Lung Cancer Screening in Medicare Patients The graph represents the cumulative percentage of 7292 enrollees aged 55 to 80 years who participated in SDM in 2016 or 2017 and who subsequently underwent low-dose computerized tomographic (LDCT) screening by month after SDM. Of these 7292 patients who had an SDM visit in 2016 and 2017, 5081 (69.7%) received LDCT within 12 months. Of the enrollees receiving LDCT, 4652 (91.6%) underwent LDCT within 3 months of the SDM visit.

**Table.** Observed Rates and Adjusted Odds of Receiving LDCT Lung Cancer Screening Within 90 Days After Medicare Enrollees Had a Visit for SDM

Characteristic	Patients, No.		LDCT rate, % (95% CI)	OR (95% CI)^a^	
	SDM (n = 11 699)	LDCT (n = 7522)		Model 1	Model 2
Overall			64.3		
Age, y					
≥55 to ≤65	2772	1795	64.8 (62.9-66.5)	1 [Reference]	1 [Reference]
>65 to ≤70	4953	3329	67.2 (65.9-68.5)	1.10 (0.97-1.25)	1.10 (0.97-1.25)
>70 to ≤75	3217	2005	62.3 (60.6-64.0)	0.93 (0.81-1.06)	0.94 (0.82-1.07)
>75 to ≤80	757	393	51.9 (48.3-55.5)	0.69 (0.57-0.85)	0.70 (0.57-0.86)
Year					
2016	2632	1633	62.0 (60.2-63.9)	1 [Reference]	1 [Reference]
2017	4660	3019	64.8 (63.4-66.2)	1.13 (0.99-1.28)	1.10 (0.97-1.24)
2018	4407	2870	65.1 (63.7-66.5)	1.14 (0.99-1.30)	1.11 (0.98-1.26)
Gender					
Male	5893	3884	65.9 (64.7-67.1)	1 [Reference]	1 [Reference]
Female	5806	3638	62.7 (61.4-63.9)	0.87 (0.80-0.95)	0.87 (0.79-0.95)
Medicaid					
No	9183	5964	65.0 (64.0-65.9)	1 [Reference]	1 [Reference]
Yes	2516	1558	61.9 (59.9-63.8)	0.91 (0.81-1.03)	0.91 (0.80-1.02)
Race/ethnicity					
Non-Hispanic White	10 639	6650	64.1 (63.2-65.1)	1 [Reference]	1 [Reference]
Black	710	447	63.0 (59.3-66.5)	0.87 (0.72-1.06)	0.87 (0.72-1.06)
Hispanic	229	144	62.9 (56.3-69.2)	0.86 (0.62-1.20)	0.78 (0.56-1.09)
Other	391	281	71.9 (67.1-76.3)	1.32 (1.01-1.73)	1.26 (0.96-1.65)
Patient had seen this SDM clinician previously					
Yes	6446	3577	55.5 (54.3-56.7)	NA	1 [Reference]
No	5253	3945	75.1 (73.9-76.3)	NA	1.49 (1.34-1.03)
Clinician specialty					
Family practice	3234	1743	53.9 (52.2-55.6)	NA	1 [Reference]
Internal medicine	2480	1514	61.1 (59.1-63.0)	NA	1.18 (1.01-1.36)
Pulmonary	1810	1021	56.4 (54.1-58.7)	NA	0.84 (0.70-1.01)
Radiologist	172	160	93.0 (88.1-96.3)	NA	9.09 (4.16-19.85)
Nurse practitioner	2738	2193	80.1 (78.6-81.6)	NA	1.70 (1.42-2.05)
Physician assistant	734	532	72.5 (69.1-75.7)	NA	1.40 (1.08-1.80)
Other	531	359	67.6 (63.4-71.6)	NA	1.27 (0.97-1.66)

Abbreviations: ICC, intraclass correlation coefficient; LDCT, low-dose computerized tomographic; NA, not applicable; OR, odds ratio; SDM, shared decision-making.

^a^ Model 1 includes patient characteristics and Model 2 adds clinician characteristics from a 2-level hierarchical generalized linear mixed model (enrollee and clinician). ICC for model 1 was 27.42%; ICC for model 2 was 24.70%. The adjusted odds of receiving LDCT and 95% CIs were calculated from a 2-level hierarchical generalized linear mixed model with the clinician as a random effect. The ICCs estimated the outcome associated with adding the clinician.^6^

The highest rates of subsequent LDCT screenings were following SDM visits with radiologists (93.0%; 95% CI, 88.1%-96.3%), followed by nurse practitioners (80.1%; 95% CI, 78.6%-81.6%), with lower rates from pulmonary specialists (56.4%; 95% CI, 54.1%-58.7%) and family physicians (53.9%; 95% CI, 52.2%-55.6%). The adjusted odds ratio of undergoing LDCT screening after an SDM with a radiologist was 9.09 (95% CI, 4.16-19.85); a nurse practitioner, 1.70 (95% CI, 1.42-2.05); and a pulmonary specialist, 0.84 (95% CI, 0.70-1.01) (family physicians were used as a reference for odds ratios). Approximately 55% of enrollees (6446 of 11 699 [55.1%]) had seen the SDM clinician in the year prior to the SDM visit. In these patients, the rate of LDCT was 55.5% (95% CI, 54.3%-56.7%) vs 75.1% (95% CI, 73.9%-76.3%) in those who had not previously seen the clinician.

The intraclass correlation coefficient (ICC) for model 1 in the Table was 27.4%, indicating that 27% of the variation in whether patients received LDCT after SDM was associated with which clinician they saw.

## Discussion

The decision to undergo LDCT lung cancer screening varies substantially by the clinician participating in the SDM. Ideally in SDM, one might expect that all the variation in choice of LDCT would be because of differences between individual enrollees in perceived risk of cancer, willingness to undergo surgery if recommended, and personal values.

We found that SDM with a clinician with prior experience with the patient led to substantially lower rates of LDCT (55% vs 75%). Possible explanations include a greater familiarity with patient values and more trust. Some of the SDM clinicians previously unknown to the patient might have been affiliated with LDCT screening centers, which may be biased toward LDCT.^3^

This study had several limitations. No information on which clinicians were affiliated with LDCT programs and whether an SDM tool was used were available. Also, there is no ideal rate of LDCT after SDM because the decision is supposed to be based on patient-specific factors. As previously reported,^4,5^ the overall rate of SDM visits is very low, a surprising finding given that the Centers for Medicare & Medicaid Services made a separate SDM visit a requirement for reimbursement for LDCT.^2^

In this study, clinician specialty was associated with the outcome of an SDM visit. SDM with clinicians known to the patient was associated with lower LDCT rates.
